# Reconstitution and functional studies of hamster P-glycoprotein in giant liposomes

**DOI:** 10.1371/journal.pone.0199279

**Published:** 2018-06-18

**Authors:** SooHyun Park, Sheereen Majd

**Affiliations:** 1 The Brown Foundation Institute of Molecular Medicine and Texas Therapeutics Institute, University of Texas Health Science Center at Houston, Houston, Texas, United States of America; 2 Department of Biomedical Engineering, University of Houston, Houston, Texas, United States of America; Oregon State University, UNITED STATES

## Abstract

This paper describes the preparation of giant unilamellar vesicles with reconstituted hamster P-glycoprotein (Pgp, ABCB1) for studying the transport activity of this efflux pump in individual liposomes using optical microscopy. Pgp, a member of ABC (ATP-binding cassette) transporter family, is known to contribute to the cellular multidrug resistance (MDR) against variety of drugs. The efficacy of many therapeutics is, thus, hampered by this efflux pump, leading to a high demand for simple and effective strategies to monitor the interactions of candidate drugs with this protein. Here, we applied small Pgp proteoliposomes to prepare giant Pgp-bearing liposomes via modified electroformation techniques. The presence of Pgp in the membrane of giant proteoliposomes was confirmed using immunohistochemistry. Assessment of Pgp ATPase activity suggested that this transporter retained its activity upon reconstitution into giant liposomes, with an ATPase specific activity of 439 ± 103 nmol/mg protein/min. For further confirmation, we assessed the transport activity of Pgp in these proteoliposomes by monitoring the translocation of rhodamine 123 (Rho123) across the membrane using confocal microscopy at various ATP concentrations (0–2 mM) and in the presence of Pgp inhibitors. Rate of change in Rho123 concentration inside the liposomal lumen was used to estimate the Rho123 transport rates (1/s) for various ATP concentrations, which were then applied to retrieve the Michaelis-Menten constant (*K*_*m*_) of ATP in Rho123 transport (0.42 ± 0.75 mM). Similarly, inhibitory effects of verapamil, colchicine, and cyclosporin A on Pgp were studied in this system and the *IC*_*50*_ values for these Pgp inhibitors were found 26.6 ± 6.1 μM, 94.6 ± 47.6 μM, and 0.21 ± 0.07 μM, respectively. We further analyzed the transport data using a kinetic model that enabled dissecting the passive diffusion of Rho123 from its Pgp-mediated transport across the membrane. Based on this model, the permeability coefficient of Rho123 across the liposomal membrane was approximately 1.25×10^−7^ cm/s. Comparing the membrane permeability in liposomes with and without Pgp revealed that the presence of this protein did not have a significant impact on membrane integrity and permeability. Furthermore, we used this model to obtain transport rate constants for the Pgp-mediated transport of Rho123 (m^3^/mol/s) at various ATP and inhibitor concentrations, which were then applied to estimate values of 0.53 ± 0.66 mM for *K*_*m*_ of ATP and 25.2 ± 5.0 μM for verapamil *IC*_*50*_, 61.8 ± 34.8 μM for colchicine *IC*_*50*_, and 0.23 ± 0.09 μM for cyclosporin A *IC*_*50*_. The kinetic parameters obtained from the two analyses were comparable, suggesting a minimal contribution from the passive Rho123 diffusion across the membrane. This approach may, therefore, be applied for screening the transport activity of Pgp against potential drug candidates.

## Introduction

This paper applies a liposome-based assay for monitoring the transport activity of a multidrug resistance (MDR) transporter, P-glycoprotein (Pgp, ABCB1) from hamster, at a single-liposome level using confocal microscopy. Pgp is a 170 kDa transmembrane efflux pump from the ATPase-binding cassette (ABC) protein family [1–4]. Pgp consists of 12 transmembrane (TM) domains assembled as dimer-like halves, with its substrate-binding sites located within its TM domain and its ATP-binding cassettes located on its cytosolic domain [3, 5, 6]. With an ATP-driven conformational change, Pgp is known to bind to and transport a broad range of amphiphilic substrates out of cells [4, 7–10]. Normally, Pgp plays a crucial role in clearance of foreign molecules and toxins from organs, which explains its presence in digestive system, central nervous system, and placenta [3, 11, 12]. However, this protein is also present in tumors, where its interactions with therapeutic agents often lead to reduction in their intracellular concentrations and hamper their effectiveness [13]. In cancer treatment, therefore, this protein is believed to be a major contributor to the multidrug resistance developed in malignant cells [13–16]. In fact, Food and Drug Administration (FDA) requires careful screening of candidate drugs against Pgp during the drug development stages to provide reliable predictions of their *in vivo* therapeutic efficacy [17]. Much effort has, thus, been focused on studying Pgp structure and function in healthy and diseased conditions [4, 6, 14, 18, 19]. Most of the Pgp substrates are small and amphiphilic and can diffuse freely across the membrane. Upon recognition of its substrates, either in the cytoplasm or inner leaflet of bilayer, and using the energy driven from the ATP hydrolysis, Pgp undergoes a conformational change, transporting its substrates out of the cell [4]. While studies on Pgp crystal structure have revealed multiple substrate binding sites in the transmembrane domain of this protein [6, 18, 20], its exact mechanism of substrate transport remains unclear [4, 21, 22], hindering the development of effective strategies to inhibit or bypass Pgp during treatment of diseases such as cancer.

Most of the previous studies on Pgp have focused on biochemical characterization of this pump in cell cultures [4]. However, the literature results often vary widely by specific cell lines and the experimental conditions. One of the strategies that can reduce this discrepancy is the reconstitution and studying of Pgp in liposomal membranes under well-defined conditions [23]. Model membrane systems including liposomes and planar lipid bilayers offer simple platforms to study molecular transport across lipid bilayers in the absence of complexity of inner and outer cellular compartments [24–30]. Furthermore, when membrane proteins are embedded into liposomal membrane, resultant proteoliposomes enable exclusive studies on the functional aspects of these proteins [23, 31–34]. Yet, current liposome-based approaches mostly employ nano-scale liposomes, in which the transmembrane proteins are reconstituted, and rely on ensemble of data from populations of these small liposomes [31, 35, 36]. More recently, these approaches have been further extended to large and giant liposomes that allow for functional studies of proteins, including Pgp and G-proteins, at a single-liposome level [37–41]. Micron-scale liposomes are more comparable to natural cell membranes and more importantly, can be resolved under optical microscopy. In this study, we reconstitute hamster Pgp to giant liposomes using electroformation, the most common technique for the preparation of giant liposomes. To the best of our knowledge, this is the first report on functional reconstitution of an ABC transporter into giant liposomes via electroformation. We then apply giant liposomes with reconstituted hamster Pgp to monitor the transport activity of this pump in individual liposomes under various conditions and to determine the corresponding kinetic parameters.

Herein, we apply Pgp bearing giant liposomes (diameters ≥ 10 μm) prepared by a modified form of electroformation, in which aqueous dispersion of small proteoliposomes are deposited or stamped onto indium tin-oxide (ITO) plates and applied for electroformation of giant proteoliposomes [30, 42]. This approach enables easy manipulation of lipid composition on giant proteoliposomes without additional reconstitution steps, minimizing the consumption of purified Pgp. Using confocal microscopy, we directly monitor translocation of a Pgp fluorescent substrate, rhodamine 123 (Rho 123), across the liposomal membrane at different ATP concentrations and in the presence of three Pgp inhibitors, verapamil, colchicine, and cyclosporin A. We apply this assay to study the kinetics of Pgp-mediated Rho123 transport and report the Michaelis-Menten constant (*K*_*m*_) for ATP in Rho123 transport as well as *IC*_*50*_ values for verapamil, colchicine, and cyclosporin A. By comparing the results and the estimated kinetic parameters to those previously reported from biochemical assays, we demonstrate the reliability of the present giant proteoliposome-based assay for Pgp transport kinetic studies.

## Materials and methods

### Materials

1-palmitoyl-2-oleoyl-*sn*-glycero-3-phosphocholine (POPC), 1,2-dioleoyl-*sn*-glycero-3-phosphoethanolamine-N-(biotinyl) (biotinyl-PE), 1,2-dipalmitoyl-*sn*-glycero-3-phosphoethanolamine-N-(lissamine rhodamine B sulfonyl) (rho-PE), 1,2-diphytanoyl-*sn*-glycero-3-phosphoethanolamine-N-(7-nitro-2-1,3-benzoxadiazol-4-yl) (NBD-PE), and cholesterol were purchased from Avanti Polar lipids, Inc (Alabaster, AL). Purified Pgp extracted from Chinese Hamster Ovary B30 cells and some of Pgp reconstituted small liposomes (referred to as small proteoliposome-A or small PL-A in the manuscript, see **Table 1** for lipid composition) were generous gifts from Dr. Frances Sharom’s lab (Molecular Cellular Biology, University of Guelph, Ontario, Canada). HEPES buffer (20 mM HEPES, 5 mM MgCl_2_, 100 mM NaCl pH 7.5), Sephadex G50, streptavidin, ATP, Rho123, verapamil, colchicine, cyclosporin A, and rhodamine B isothiocyanate-Drxtran (70,000 MW) (RITC-Dextran) were from Sigma-Aldrich (Saint Louis, MO). 3-[(3-cholamidopropyl)dimethylammonio]-1-propanesulfonate (CHAPS) was from MP Biomedicals Inc. (Santa Ana, CA), and creatine kinase and creatine phosphate were from Roche (Basel, Switzerland). Mouse Pgp antibody (MDR/ABCB1 antibody C219) was purchased from Novus (Saint Charles, MO), and Alexa Fluor^®^ 488 anti-mouse secondary antibody was from Thermo Fisher Scientific (Waltham, MA). 26G needle was from Becton Dickinson (Franklin Lakes, NJ).

**Table 1:** Lipid composition of the examined liposomes.

Liposome Formulation	POPC Mol %	Cholesterol Mol %	Rho-PE Mol %	NBD-PE Mol%	Biotinyl-PE Mol%	Pgp
A	99.0	-	-	1.0	-	+
B	68.8	30.0	0.2	-	1.0	+
C	99.0	-	1.0	-	-	-
D	48.8	50	0.2		1.0	-
E	61.8	37.0	0.2	-	1.0	+

### Reconstitution of P-glycoprotein in small liposomes

Table 1 summarizes the lipid composition of all the liposomal formulations used in this study. Proteoliposomes of various formulations will be referred to as proteoliposome-formulation (e.g. PL-B) throughout the manuscript. For small PL-Bs that were prepared in our laboratory, we followed the protocol previously described by Sharom and colleagues [23, 33]. Specifically, purified Pgp in 500 mM CHAPS (in HEPES buffer) was reconstituted into small liposomes at a 10:1 (lipid: Pgp) weight ratio via detergent removal method. First, small unilamellar vesicles (SUVs) with a lipid composition A or B (see Table 1) were prepared in 500 mM CHAPS in HEPES buffer. The SUV solution and Pgp in CHAPS were then mixed together and passed through a 26G needle repeatedly. Then, we ran the mixture through a Sephadex G50 column and collected fractions at a flow rate of ~ 1 drop/ 4 seconds. The protein concentration on each fraction was measured using a Bradford assay (Bio-rad, Hercules, CA). After reconstitution, small PL-Bs were stored at -80 ^o^C until usage, and the samples were used within a year of reconstitution.

### Electroformation of P-glycoprotein bearing giant liposomes

For electroformation of giant proteoliposomes, aqueous dispersion of small liposomes with reconstituted Pgp were deposited on ITO plates either directly [42–45] or using our previously reported method of hydrogel-stamping; both methods have shown successful functional reconstitution of membrane proteins [30, 39]. Hydrogel-stamping of lipid/proteins, which enabled us to control the size of giant proteoliposomes, was preferred for samples with higher protein/lipid concentrations (e.g. small PL-A with 3 mg/mL lipid concentration) that produced relatively thick lipid/protein deposits. An agarose hydrogel stamp was used to pattern lipids/proteins onto ITO, where the deposits were partially dried for up to 1 hour at room temperature. Complete dehydration of lipid/protein deposits was avoided to better preserve the activity of Pgp. Next, a fluidic chamber was assembled by sandwiching a thin PDMS (polydimethylsiloxane) frame with tubing connections, between two patterned ITO plates. Stamped deposits were rehydrated in the fluidic chamber using a 235 mM sucrose solution containing a large hydrophilic fluorescent dye, RITC-Dextran, and were subjected to an AC current (2 p-p V, 50 Hz) for 2 hours on ice to protect the protein activity. Resultant giant proteoliposomes were detached by reducing the frequency to 1 Hz for ~ 30 min. To assess the integrity of giant liposomal membrane, outside solution containing RITC-Dextran was washed by a 235 mM glucose solution (note that osmolality was matched to the inside solution, 235 mM sucrose), and the complete dye encapsulation was confirmed under the confocal microscopy. Alternatively, for a higher yield of giant proteoliposomes when working with low lipid/protein concentrations, lipid/protein deposits were produced by direct deposition of small proteoliposomes (e.g. PL-B) solution on the ITO. In this approach, upon partial dehydration, the lipid/protein deposits were subjected to a 5 p-p V and 50 Hz frequency for 2 hr on ice in 235 mM sucrose. Upon detachment, the giant proteolipoosomes were harvested and immediately used for the following experiments.

**Fig 1:**
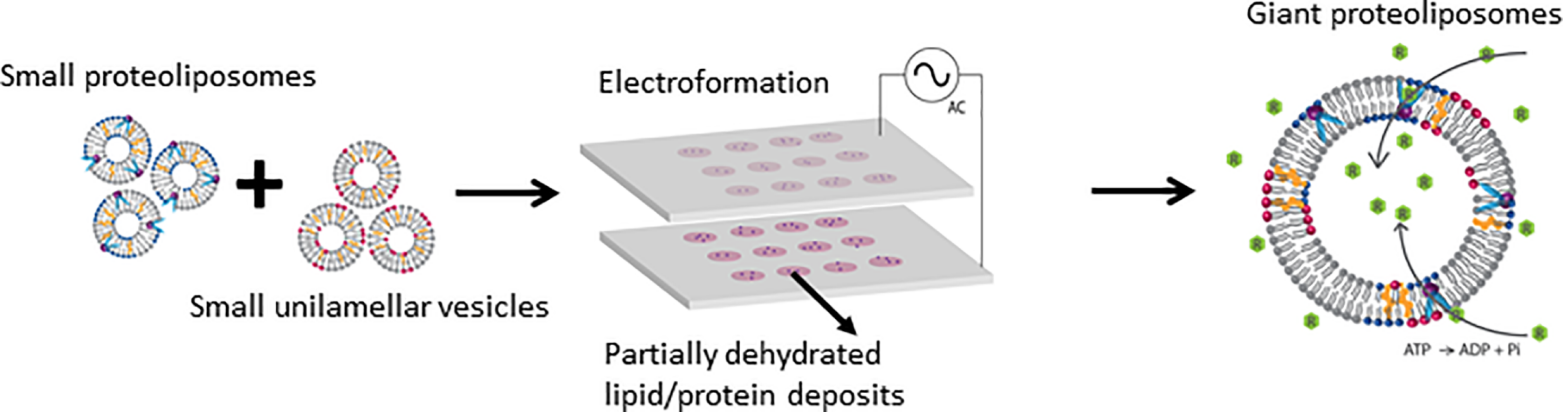
Preparation of giant proteoliposomes. Cartoon illustration of the concept applied for the formation of Pgp-bearing giant liposomes from small proteoliposomes with control over the final lipid composition of the giant liposomes. First, small proteoliposomes with reconstituted Pgp were mixed with small liposomes of a distinct composition, and the mixture was deposited onto ITO plates and partially dried. Upon rehydration of the partially dried lipid/protein films, an AC current was applied to electroform giant Pgp-bearing liposomes with the desired lipid composition. The resultant giant proteoliposomes were used for studying transport activity of Pgp by monitoring translocation of a fluorescent Pgp substrate, Rho123 (shown as green R molecules) across membrane, upon Pgp activation by ATP hydrolysis.

Besides direct application of small proteoliposomes to electroform giant proteoliposomes of similar composition, we applied mixtures of small proteoliposomes with SUVs of different compositions, prior to electroformation, to tune the lipid composition of resultant giant proteoliposomes (Fig 1). To verify whether the mixture of small liposomes with different lipid compositions would be incorporated in the resultant giant liposomes, various volume mixtures of Pgp-bearing small liposomes containing green fluorescence tagged lipid (NBD-PE) and SUVs with red fluorescence tagged lipid (Rho-PE) were deposited onto the ITO plates and subjected for electroformation. Red and green fluorescence intensities from each small liposomes were measured in the resulted giant liposomes to confirm the respectful incorporation of lipids. Following this approach, a 2:1 (v/v) mixture of PL-B and SUV-D was applied for electroformation of giant PL-E with higher cholesterol content compared to the small proteoliposomes. The increased cholesterol content in the membrane was expected to improve the integrity of the membrane [46, 47].

### Immunostaining against Pgp antibody on giant liposomes

In order to confirm the presence of Pgp on liposomal membranes after the electroformation process, giant proteoliposomes were stained against the Pgp antibody. Specifically, the giant PL-Es in 235 mM sucrose were harvested after electroformation and reseeded on a coverslip. This coverslip was coated with streptavidin (2 hour incubation with 82 nM streptavidin solution) to enable anchoring the biotinylated liposomes onto the surface. It should be noted that non-coated areas of the coverslip were blocked with a 1 mg/mL BSA solution to prevent rupture of liposomes. Next, the fluid chamber was filled with a 235 mM glucose solution to facilitate the sedimentation of liposomes; this was achieved due to the density difference between the two sugar solutions while the osmolality inside and outside liposome was matched. Upon docking the liposomes on the surface, a solution of 1:20 dilution of the mouse anti-Pgp in HEPES buffer was introduced to the chamber and incubated overnight at 4 ^o^C. The sample was next washed with the HEPES buffer at a flow rate of 1 mL/ hr and incubated with the secondary antibody, Alexa Fluor^®^ 488 anti-mouse (1:250 dilution in the HEPES buffer) at the room temperature for 2 hours. Lastly, the antibody solution was replaced with the HEPES buffer, and the immunostained giant liposomes were imaged using an inverted confocal microscope (Olympus FV1000, Tokyo, Japan). As a negative control, the giant liposomes were incubated only with the secondary antibody (1:250 dilution in the HEPES buffer) and imaged under identical conditions as the positively stained liposome.

### ATPase activity assay

Functionality of Hamser Pgp on giant proteoliposomes was evaluated by a colorimetric ATPase activity assay, following a previously published protocol [23]. Here, we prepared giant PL-B to maximize the number of Pgp on giant liposomes for a population-based assay, and 20 μL of harvested giant PL-B samples were subjected to the ATPase activity assay. Briefly, Pgp molecules on the giant liposome were activated by ATP introduction. As Pgp hydrolyzed the ATP, inorganic phosphate was produced as a byproduct. The reaction was stopped by addition of a solution of 6% SDS (sodium dodecyl sulfate), 3% ascorbic acid, and 0.5% ammonium molybdate in 0.5 M HCl. Then, a solution of 2% sodium arsenite, 2% sodium citrate, and 2% acetic acid was added to develop the color with an intensity corresponding to the phosphate ion concentration in the solution. Lastly, the absorbance at 750 nm was measured. The ATPase activity was assessed at 37 ^o^C with 1 mM ATP for 30 min. For these experiments, the concentration of Pgp in the batches of small PL-B and giant PL-B samples was measured using either a Bradford assay (Biorad) or a NanoOrange Protein Quantification Kit (Thermo Fisher Scientific). The ATPase specific activity was determined by estimating the nmol of inorganic phosphate production per min per mg of protein (nmol/mg protein/ min) based on the known phosphate ion concentrations in the standards.

### Transport activity assay

Transport activity of Pgp in giant proteoliposomes was studied by monitoring the flux of a fluorescent Pgp substrate, Rho123, across the liposomal membrane in the presence of ATP and Pgp inhibitors verapamil, colchicine, and cyclosporin A. To this end, the giant PL-Es were harvested from electroformation chamber and reseeded on a streptavidin-coated cover slip for less than 10 min on ice. Upon sedimentation and docking of liposomes onto the surface through biotin-streptavidin binding, a transport cocktail containing Rho123 (fixed at 525 nM), ATP (0–2 mM), ATP regenerating system (creatine kinase and creatine phosphate), and/or Pgp inhibitors (verapamil, colchicine, or cyclosporin A) in HEPES buffer was quickly introduced to the liposome containing chamber. The liposome inside solution (235 mM sucrose) had a matching osmolality to that of HEPES buffer of transport cocktail, to eliminate osmotic pressure across the membrane. Single giant liposomes were then promptly located under the inverted confocal microscope at 60x magnification using epi-fluorescence. Next, we collected time-lapse confocal images of the liposome using 488 nm and 543 nm lasers, to image Rho123 (inside liposome) and Rho-PE (on membrane) respectively. These images were collected at 30–90 second intervals for 20–45 min. At occasions where an individual liposome was not located at early time points, the image acquisition started at later time points as long as the liposome was not already filled with Rho123 to the level that the intensity inside and outside the liposomes were indistinguishable; the lower fluorescence intensity inside the liposome indicated that the active transport was in action. All imaging was performed under identical acquisition settings (i.e. laser power, scanning speed, HV, offset, and gain).

### Data analysis of P-glycoprotein transport activity with rhodamine123

Rho123 fluorescence intensity inside and outside of giant proteoliposomes was analyzed using ImageJ. Liposome diameters were also measured using ImageJ based on the rho-PE signal outlining the liposomal membrane. Background Rho123 intensity inside the liposome at time 0 (*I*_*inside t = 0*_) was subtracted from all the measured Rho123 intensities for time ≥ 0 (i.e. *I*_*inside*_ and *I*_*outside*_) in order to take account for Rho123 signal from the out of focus planes. In cases where the data collection started after time 0, the linear regression was performed to estimate Rho123 intensity inside the liposome at *t* = 0 assuming that the rate of change in fluorescence intensity was constant for early time points. A standard curve of Rho123 intensity as a function of its concentration was confirmed the linear correlation within the concentration range used. This standard curve was used to convert the measured fluorescence intensity values to the Rho123 concentration inside the giant liposomes. The concentration of Rho123 inside the liposome (*C*_*i*_) was then normalized to its outside concentration (*C*_*o*_) at each time point in order to account for possible signal fluctuations during time-lapse imaging. The described background subtraction and normalization for each time point is summarized in Eq 1 below.

$$\left(\frac{I_{inside}-I_{inside\;t=0}}{I_{outside}-I_{inside\;t=0}}\right)\propto \frac{C_{i}}{C_{o}}$$ (1)

The normalized Rho123 concentration (*C*_*i*_*/C*_*o*_) inside each liposome was plotted against time for further assessment of Rho123 influx rate. The slope of the linear portion of this curve was determined and used as the rate of Rho123 influx (1/s) for individual vesicles. The collected transport rates were plotted as a function of ATP concentration, and *K*_*m*_ value was estimated by fitting the data into Michaelis-Menton (M-M) equation using Prism® (Graphpad Software, La Jolla, CA). The transport rate was also plotted against inhibitor concentrations, and the half maximal inhibitory concentration (*IC*_*50*_) of each inhibitor was determined by fitting the data to a one-phase exponential decay model.

In order to further break down the Pgp transport activity, and to ensure that the passive diffusion of substrate across the membrane did not significantly contribute to the observed influx, we fitted *C*_*i*_*/C*_*o*_ over time into a model, adapted from the previous work of Horger *et al* [38], which allowed for dissection of the Pgp-mediated transport and diffusion. By fitting the data into this model using a least-squares fit algorithm in MATLAB^®^, we retrieved two transport kinetics parameters of membrane permeability coefficient, *P*_*s*_, and transport rate constant, *k*, from the change in Rho123 concentration recorded during the Pgp transport activity in individual liposomes. Transport rate constants plotted against ATP concentration were fitted to Michaelis-Menten model to determine *K*_*m*_. Also, transport rate constants as a function of Pgp inhibitor concentration (i.e. verapamil, colchicine, and cyclosporin A) were fitted to a one-phase exponential decay model to determine *IC*_*50*_ values for each of these inhibitors.

## Results and discussions

In order to prepare Pgp-bearing giant liposomes, we first reconstituted Pgp into small liposomes and then applied these liposomes for electroformation. To this end, we used purified Pgp, extracted from Chinese Hamster Ovary B30 cells and applied size exclusion chromatography to reconstitute these proteins at a lipid to Pgp ratio of 10:1 (w/w) in desired liposome formulation (see Table 1 for the lipid formulations). Using the Bradford assay, we found that Pgp concentration after reconstitution was 0.14 mg/mL suggesting an approximately 70% loss in protein mass during the reconstitution and/or detergent extraction steps. Assuming that the lipid to Pgp ratio remained unchanged during the reconstitution process, this result suggested a final lipid concentration of 1.4 mg/mL in the liposomal solution. This protein mass recovery rate was higher than the reported value of ~ 10% for human Pgp liposomal reconstitution, but lower than 45–55% recovery rate previously reported for hamster Pgp in nano-scale liposomes [38, 48–50].

Aqueous dispersion of small Pgp proteoliposomes was then deposited or stamped onto ITO plates and applied for electroformation to prepare giant proteoliposomes as detailed in the experimental section. Electroformation protocol was carefully adjusted in order to preserve functional activity of Pgp. Complete dehydration of lipid/protein deposits was avoided though there was significant loss of lipid/protein during rehydration. Also, once rehydrated, Pgp containing chamber was kept on ice until the ATPase or transport activity assays, even during the electroformation process. Hydrogel stamping of the liposome dispersions enabled production of uniformly-sized giant liposomes; the diameter of giant PL-A was 36.55 ± 8.59 μm. However, direct deposition of liposome dispersions was preferred when the concentration of small proteoliposomes were low (e.g. small PL-B).

**Fig 2:**
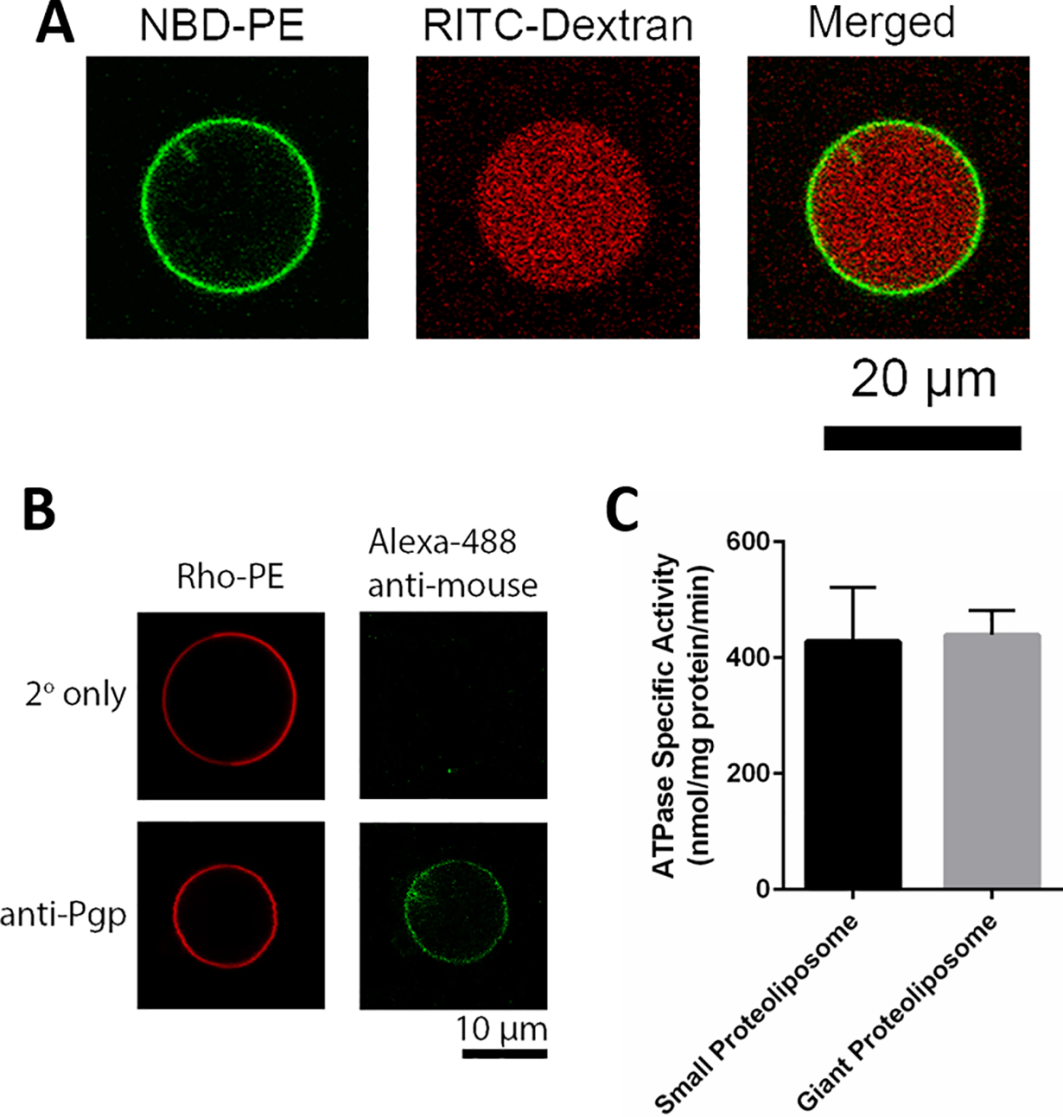
Immunostaining and ATPase activity of Pgp on giant liposomes. (A) Confocal images of an electroformed giant PL-A. Green signal is from NBD-PE in the membrane and red signal is from RITC-Dextran, encapsulated in the liposomal lumen. (B) Immunostaining of a giant PL-C against anti-Pgp antibody. Positive staining (bottom panels) against anti-Pgp is shown with green signal with the secondary antibody, Alexa Fluor® 488 anti-mouse, and liposome membrane was labeled with rho-PE in red. The negative control (top panels) was performed on similar liposomes in the absence of the primary antibody incubation. (C) ATPase specific activity of Pgp in similarly formulated small PL-B and giant PL-B. The bars represent the mean and error bars show standard error of mean (SEM) with n = 3–6, from independent electroformation events.

Membrane integrity in the resultant giant proteoliposomes were assessed by monitoring the encapsulation of a large hydrophilic dye (RITC-Dextran 70,000 MW) in these liposomes. Upon introduction of this dye during the electroformation, which led to its entrapment in liposomal lumen, the liposome outside solution was exchanged to remove the untrapped dye. As shown in Fig 2A, the giant proteoliposomes were able to retain the encapsulated RITC-Dextran, which confirmed their membrane integrity.

The lipid composition in the electroformed giant liposomes was not limited to that of the small proteoliposomes. By mixing the small proteoliposomes with other liposomal formulations prior to electroformation, we were able to fine-tune the lipid composition of the electroformed giant proteoliposomes without the need for multiple reconstitution steps (Fig 1). For instance, we electroformed mixtures of small PL-A and SUV-C and were able to detect both fluorescence signals (green, from NBE-PE of small PL-A and red, from rho-PE of SUV-C) on the resultant giant proteoliposomes, suggesting that mixing different populations of small liposomes before electroformation enabled control over the composition of giant proteoliposomes (S1 Fig). Furthermore, when we varied the volume ratios of the small PL-A and SUV-C in the mixture applied for electroformation, the red and green fluorescence signal on the resulting giant proteoliposomes varied accordingly (S1 Fig). We, therefore, applied this strategy to control the lipid composition of the giant liposomes during the transport activity assay.

In order to confirm the presence of Pgp on these giant liposomes, we applied immunostaining against Pgp antibody. Giant PL-Bs with a red fluorescent-labeled membrane were incubated with an anti-Pgp antibody followed by a green fluorescent mouse secondary antibody. As displayed in Fig 2B, binding of the secondary antibody to these liposomes confirmed the presence of Pgp on their membranes. A control giant PL-B exposed only to the secondary antibody (without primary antibody incubation) did not show a detectable green fluorescence signal, confirming the binding specificity.

Next, we assessed the ATPase activity of Pgp in giant liposomal membranes (giant PL-B) as a measure of its functionality [23, 51]. To this end, we used an ATPase activity assay that measures the amount of inorganic phosphate produced during the ATP hydrolysis by Pgp. ATP was introduced to the 96-well plate containing Pgp-bearing giant liposomes, where it could only activate outward Pgp molecules (i.e. with their nucleotide binding domains exposed to the liposome exterior). Note that we assumed that ATP did not cross the membrane and thus, did not activate the inward Pgp molecules. Level of ATPase activity of Pgp in these liposomes is presented as ATPase specific activity, which represents the amount of produced phosphate in nanomol per mass of protein in mg per time in min. As shown in Fig 2C, these measurements revealed that the average ATPase specific activity of Pgp in giant PL-Bs was 439 ± 103 nmol/mg protein/min. This value was comparable to the specific activity level of 428 ± 230 nmol/mg protein/min measured in our small PL-B samples, suggesting that the Pgp activity was not significantly affected by the electroformation process. Although this specific activity level in small PL-Bs is not as high as some of the reported values for hamster Pgp (1–2 μmol/mg protein/min) [4], presumably due to the long (>1 yr) storage of this purified protein, it is higher than that of other commercially available Pgp reconstituted systems, including mouse Pgp and human Pgp (with 43 and 67 nmol/mg protein/min, respectively) [37, 52, 53]. These results show that the electroformation method applied here, did not affect the ATPase activity of hamster Pgp and is, hence, suitable for reconstitution of Pgp in giant vesicles.

**Fig 3:**
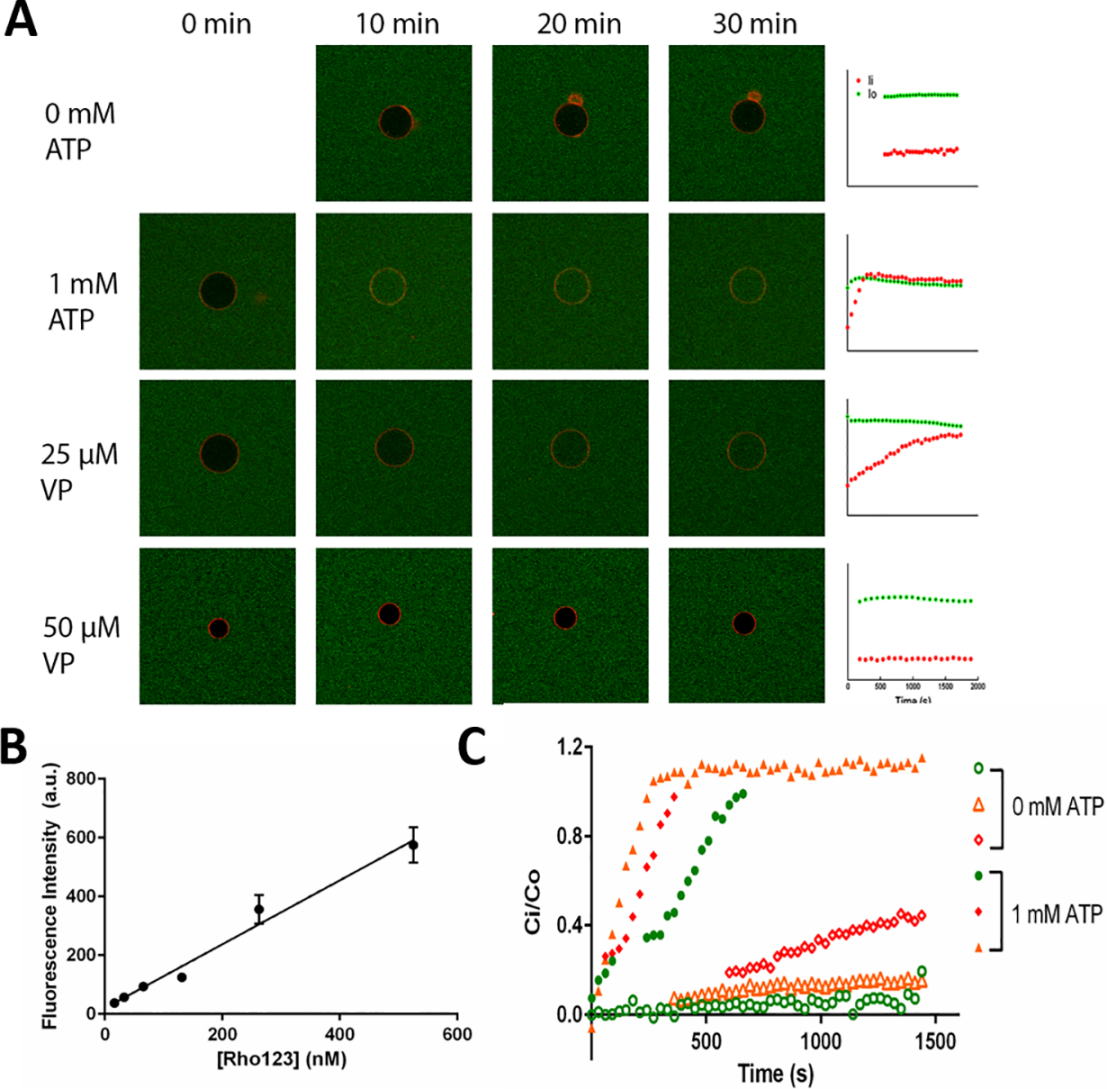
Pgp-mediated transport of Rho 123 in giant liposomes. (A) Confocal images of representative giant liposomes with PL-C membrane composition (labeled red with rho-PE) in a bath of Rho123 (green) solution. For all conditions except 0 mM ATP, transport cocktails (ATP, ATP regenerating system, and/or verapamil (VP)) were introduced at 0 min along with 525 nM Rho123. Scatter plots on the right represent the time-dependent changes in fluorescence intensity of Rho123 inside (red) and outside (green) of the giant PLs. Scale bar is 20 μm. (B) The standard curve of Rho123 fluorescence intensity versus its concentration in the fluidic chamber. Fitting of data to linear regression showed a good correlation between fluorescence intensity and concentration (y = 1.09x + 20, R^2^ = 0.96). The data points show the means and error bars represent SEM with n = 4–5 from randomly selected regions within the fluid chamber. (C) Representative scatter plots of the normalized Rho123 concentration inside the giant PL-C lumen (*C*_*i*_*/C*_*o*_) with and without ATP as a function of time. The color filled symbols represent the active transport activity with 1 mM ATP and the hollow symbols represent the negative control experiments in the absence of ATP.

In order to further evaluate the functionality of hamster Pgp in the produced giant liposomes, we monitored the translocation of a fluorescent Pgp substrate, Rho123, across the liposomal membranes. Pioneered by Sharom and her colleagues, fluorescent substrates of Pgp have been previously applied to assess the transport activity of Pgp in liposome-based assays [9, 23, 54]. Amongst fluorescent Pgp substrates, Rho123 has a reasonably high affinity for Pgp (*K*_*d*_ of 12.8 μM) and more importantly, is hydrophilic compared to other examined fluorescent Pgp substrates such as tetramethylrhosamine (TMR), Hoechst 33342, and LDS-751 [23]. Translocation of this compound can, therefore, be effectively used as a measure of Pgp transport activity with minimal contribution from its passive diffusion across the lipid membrane. Here, we probed Rho123 translocation across the membrane in individual liposomes using confocal microscopy. We first optimized the lipid composition of giant liposomes to improve membrane integrity and minimize the leakage of Rho123. Given the known positive effect of cholesterol on membrane integrity in PC membranes (up to 60 weight %) [46, 47] and its minimal impact on Pgp activity (up to ~40 mol %) [55], we modified the lipid composition of liposomes to contain 30–37 mol % cholesterol and evaluated the membrane permeability. This composition adjustment was achieved by mixing the small Pgp-bearing liposomes (with 30% cholesterol content) with small liposomes of higher cholesterol contents (e.g. 50%) to reach the desired lipid formulation. The aqueous dispersion of the mixed liposomes were then deposited onto ITO and used for electroformation. While at 30% cholesterol content, the membrane of Pgp bearing giant liposomes was relatively leaky, at 37% cholesterol content (giant PL-E), passive diffusion of Rho123 across the membrane was minimal (Fig 3A top panel). This formulation was hence, selected for the next experiments. It is noteworthy that the applied method for giant proteoliposome preparation here enabled easy manipulation of the final lipid composition in these liposomes by simply mixing the small proteoliposomes with small liposomes of distinct lipid formulation, eliminating the need for repetitive reconstitution steps.

Lamellarity of the liposome was a crucial factor to consider during the transport activity assay because multi-lamellar liposomes could lead to a false negative activity by Pgp. As others and we have previously demonstrated the technique employed in this study produces a good number of unilamellar vesicles and the large size of these liposomes allows for assessment of their lamellarity using phase-contrast and fluorescence microscopy [30, 38, 43]. To further confirm the unilamellarity of Pgp-bearing liposomes that were applied for the transport activity studies, we compared the Rho-PE fluorescence intensity of these liposomes with that of (1) giant unilamellar vesicles (GUVs) with similar lipid composition but without Pgp, and (2) multi-lamellar liposomes of similar composition without Pgp. As shown in S2 Fig, Pgp-bearing liposomes had a similar level of fluorescence intensity as the control GUVs and a significantly lower intensity than multi-lamellar liposomes, indicating that Pgp-bearing liposomes were indeed unilamellar. Next, ATP was introduced into the liposome chamber to activate Pgp, and the level of fluorescence intensity inside and outside of liposomes were recorded using time-lapse imaging under confocal microscopy. Introduction of 1 mM ATP resulted in a time-dependent increase in the liposomal lumen fluorescence intensity. This intensity change reflected an influx of Rho123 into the giant liposome presumably due to the activity of outward Pgp molecules on the membrane (Fig 3A second row). In the absence of ATP, only a slight increase in the lumen florescence intensity was detected, indicating a small contribution from the Rho123 passive diffusion across membrane during the course of the transport assay. Additionally, we were able to modulate the transport activity of Pgp by introducing its inhibitor, verapamil, to the transport cocktail. As Fig 3A bottom two rows depict, the influx of Rho123 occurred at a slower rate in the presence of 25 μM of verapamil and almost retired with 50 μM of verapamil.

To determine the transport rate of Rho123 in giant proteoliposomes, we first quantified the fluorescence intensities in and outside of the giant liposomes for each time-lapse confocal image collected. The fluorescence intensity inside the liposome at time 0 was used as the background signal and subtracted from all the measured intensities. Using a standard curve, we confirmed that the fluorescence intensity was linearly related to the Rho123 concentration within the examined range (Fig 3B). Subsequently, the fluorescence intensity inside the liposome at each time point was normalized against its outside intensity and the ratio was used to calculate *C*_*i*_*/C*_*o*_. Fig 3C demonstrates a collection of representative plots of normalized Rho123 concentrations in the lumen of giant liposomes as a function of time. Next, to account for the effect of exact size of each examined liposome on the Rho123 concentration in the PL lumen, values of *C*_*i*_*/C*_*o*_ were multiplied by (*d* μm /10 μm), where *d* is the diameter of the examined giant liposome and 10 μm represented the average diameter of the giant liposomes analyzed here. Note that although the surface density and activity of Pgp was assumed to be constant among giant liposomes, the concentration of Rho123 molecules transported into the liposome lumen depended on the liposomal membrane area (i.e. number of Pgp) and its lumen volume (e.g. doubling *d* in a liposome would halve its internalized substrate concentration). Finally, the initial linear slope of the time-dependent change in *C*_*i*_*/C*_*o*_ was retrieved as the Rho123 transport rate (1/s) for the corresponding experimental condition.

**Fig 4:**
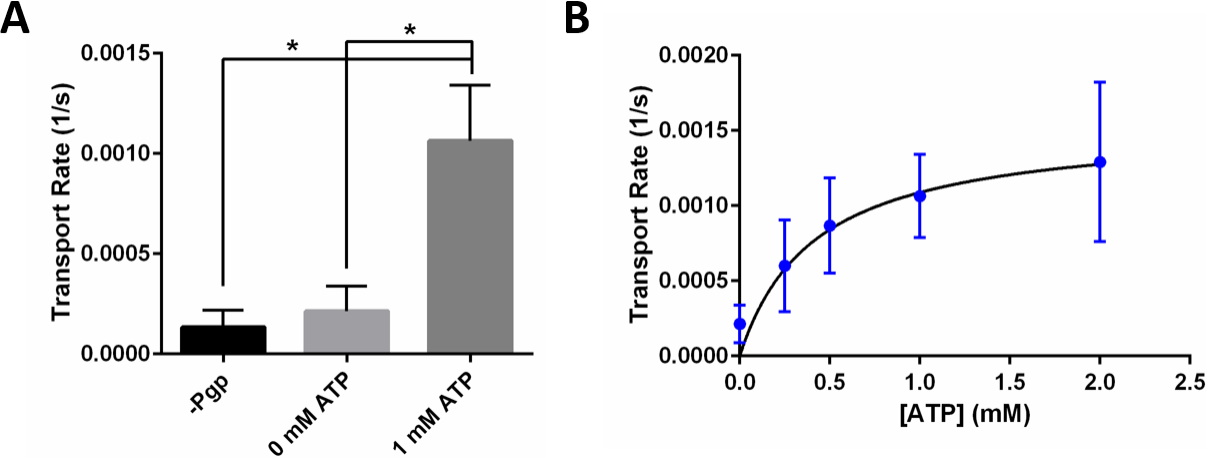
ATP dependent transport rate of Rho123 into giant liposomes by reconstituted hamster Pgp. (A) Rho123 transport rate in the presence and absence of ATP and in the absence of Pgp (–Pgp) in giant liposomes with PL-B formulation. Columns represent the mean and error bars show the SEM with n = 6–11 from independent electroformation events. * indicates p < 0.05 from non-paired t-test against 1 mM ATP transport. (B) Rho123 transport rate in giant liposomes as a function of ATP concentration. Data points represent the mean transport rate, and error bars represent SEM. The solid curve represents the best fit of the data to Michaelis-Menton model, with an estimated value of 0.42 ± 0.75 mM for *K*_*m*_ of ATP. Data points represent the mean transport rate, and error bars represent SEM from 3–11 independent electroformation events.

Fig 4A presents the retrieved Rho123 transport rates for activated Pgp compared to negative control assays. As expected, the transport rates of Rho123 in the absence of Pgp or ATP were significantly lower than that in the presence of 1 mM ATP. These data imply that ATP-driven activity of Pgp was the main source for the observed Rho123 transport into liposomes and that the Rho123 passive diffusion across the membrane did not have a significant contribution to this transport.

In order to examine Pgp transport activity as a function of ATP concentration, we measured the transport rate of Rho123 at ATP concentrations ranging from 0 to 2 mM. Changes in the rate of Pgp transport activity as a function of [ATP] can be described by the Michaelis-Menten kinetics model [23, 56, 57]:

$$v=\frac{v_{max}[S]}{K_{m}+[S]}$$ (2),

where *v* (1/s) is the reaction rate (in this case, Rho123 transport rate); *v*_*max*_ is the maximum transport rate; [*S*] (M) is the concentration of the binding substrate (in this case, ATP); *K*_*m*_ (M) represents Michaelis-Menten constant that denotes the concentration of ATP that achieves half-maximal transport rate. To apply Michaelis-Menten model to our Rho123 transport data, we assumed that the time required for ATP to bind Pgp in the membrane was negligible and that ATP concentration outside the giant proteoliposome remained constant (note that we used an ATP regenerating system, see experimental section for details). From the best fit of data to the Michaelis-Menten model, we retrieved a value of 0.42 ± 0.75 mM for *K*_*m*_ of ATP. The resultant *K*_*m*_ value is comparable to the previously reported value of 0.48 mM for hamster Pgp in small liposomes when the transport activity was assessed using another fluorescent Pgp substrate, TMR [23].

In order to further modulate the transport activity of Pgp in giant liposomes, we studied the effect of three Pgp inhibitors, verapamil, colchicine, and cyclosporin A on Pgp-mediated transport of Rho123. These drugs are known to bind to Pgp and compete with the substrate, in this case Rho123, to be transported [2, 23, 58]. For these experiments, we monitored Rho123 transport into liposomal lumen in the presence of 1 mM ATP and one of these inhibitors (at different concentration ranges based on the previous literature reports) [23, 31].

**Fig 5:**
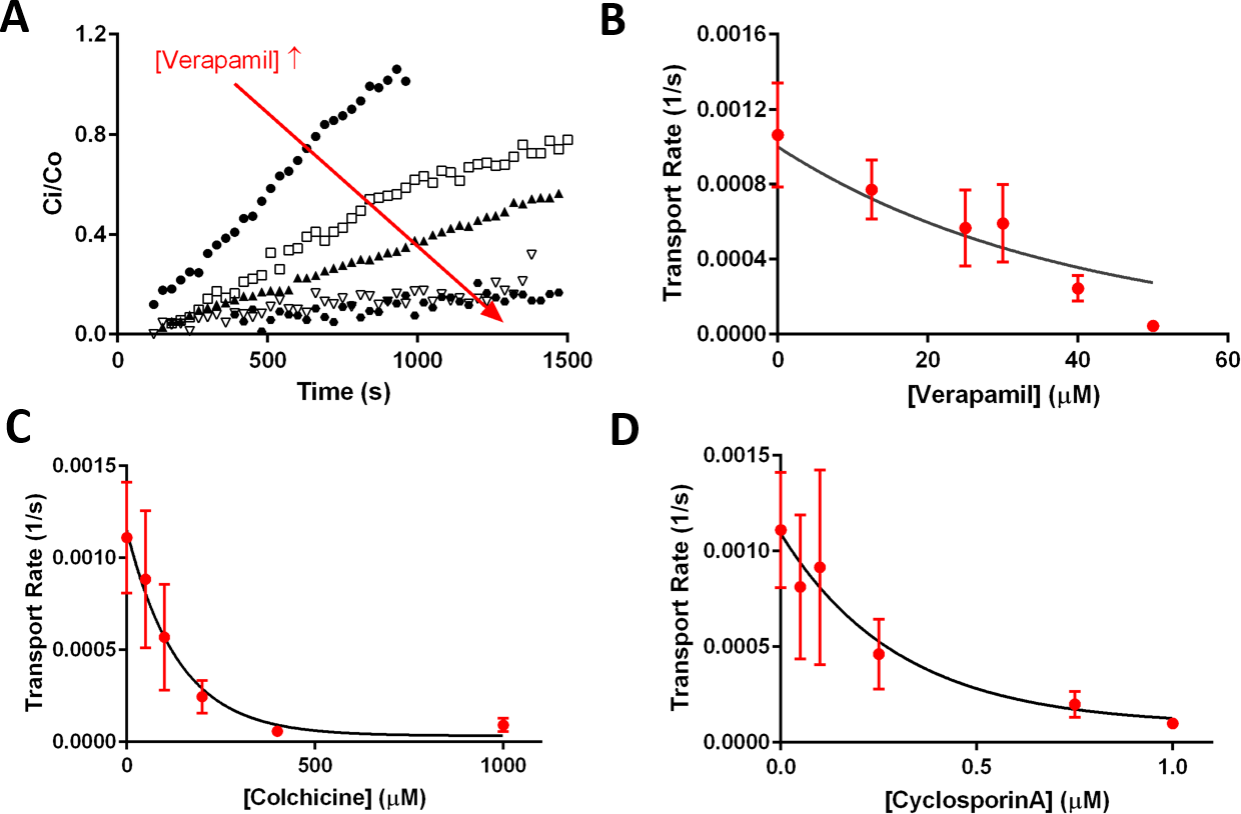
Modulation of Pgp transport activity in giant liposomes using inhibitors. (A) Representative plots of the normalized Rho123 concentration inside the giant PLs over the course of the active transport activity with increasing verapamil concentration from 12.5 μM to 50 μM. (B-D) Rate of Rho123 transport into giant PL lumen as a function of (B) verapamil concentration, (C) colchicine concentration, (D) cyclosporin A concentration. Data points represent the mean and error bars show SEM with n = 3–12 from distinct electroformation events. Data were fitted into a one-phase exponential decay model (solid curves) to estimate *IC*_*50*_ of 26.6 ± 6.1 μM for verapamil, 94.6 ± 47.6 μM for colchicine, and 0.21 ± 0.07 **μM** for cyclosporine A.

Fig 5A shows *C*_*i*_*/C*_*o*_ (i.e. ratio of interior Rho123 concentration to its concentration outside) over time with increasing concentration of verapamil from top to bottom. With an increase in the concentration of verapamil, the influx rate of Rho123 into the giant liposome reduced to reach near zero influx at 40 μM verapamil (hollow upside-down triangle on Fig 5A). To further analyze the inhibitory effect of these compounds on Pgp and determine their corresponding *IC*_*50*_ values (i.e. half-inhibition concentrations), we plotted the rate of Rho123 transport (determined as detailed earlier) into the liposomal lumen as a function of the inhibitor concentration and fitted the data to a one-phase exponential decay model as shown in Eq 3.

$$v=(v_{0}-Plateau)\times \exp\left(-k_{1}\times [inhibitor]\right)+Plateau$$ (3),

where *v*_0_ (1/s) is the maximum transport rate in the absence of an inhibitor; plateau represents the minimum transport rate; and *k*_*1*_ (1/M) is the rate of exponential decay. Based on this curve fitting, we retrieved an *IC*_*50*_ value of 26.6 μM for verapamil (Fig 5B). Using a similar approach, the *IC*_*50*_ values of colchicine and cyclosporin A were found 94.6 μM and 0.21 μM, respectively (Fig 5C and 5D). These results suggest that cyclosporin A with the smallest *IC*_*50*_ had the strongest inhibitory effect on Pgp-mediated Rho123 transport and colchicine with the largest *IC*_*50*_ had the weakest inhibitory effect on this transport. These *IC*_*50*_ values are within a reasonable range from the previously reported values of 9.1 μM for verapamil, 230 μM for colchicine, and 0.58 μM for cyclosporin A for hamster Pgp in small proteoliposomes, and exhibit a similar trend in inhibitory effect of these compounds [31].

Although *C*_*i*_*/C*_*o*_ plots in Fig 3C suggested that the passive diffusion of Rho123 across the liposomal membrane did not have a significant contribution to the observed transport of this molecule, to further dissect this transport, we adapted a model previously developed by Horger *et al*. that accounts for the contribution of passive diffusion and active transport of Rho123 by Pgp, separately [38]. On the one hand, time-dependent changes in Rho123 lumen concentration due to its passive diffusion across the membrane can be described as following, based on the Fick’s law:

$$\frac{dC_{i}}{dt}=\frac{A_{m}P_{s}}{V}(C_{o}-C_{i})$$ (4),

where *V* (m^3^) is the volume of the examined giant liposome; *P*_*s*_ (m/s) is the permeability coefficient; *A*_*m*_ (m^2^) is the membrane surface area in the giant liposome; *C*_*i*_ and *C*_*o*_ represent the inside and outside concentration of Rho123, respectively. On the other hand, time-dependent changes in Rho123 lumen concentration due to the ATP-dependent activity of Pgp can be described by:

$$\frac{dC_{i}}{dt}=\frac{k\Gamma_{Pgp}A_{m}}{V}C_{o}$$ (5),

where *k* (m^3^/mol/s) is the transport rate constant and *Γ_Pgp_* (mol/m^2^) is the surface density of Pgp on a giant liposome. Thus, the overall change in Rho123 concentration in the giant liposome can be presented as: [38]

$$V\frac{dC_{i}}{dt}=P_{s}A_{m}(C_{o}-C_{i})+k\Gamma_{Pgp}A_{m}C_{o}$$ (6)

By integrating both sides of Eq 6 (with an assumption of *C*_*i*_ = 0 at t = 0) and rearranging the resultant equation, *C*_*i*_*/C*_*o*_ can be described by the following equation.

$$\frac{C_{i}}{C_{o}}=(1-\exp\left(\frac{{-3P}_{s}t}{r}\right))(1+\frac{k\Gamma_{Pgp}}{P_{s}})$$ (7)

We used MATLAB^®^ to fit time-dependent changes in *C*_*i/*_*C*_*o*_ for each of the examined conditions into Eq 7 using a least-square fit algorithm and used these fits to determine the permeability coefficient (*P*_*s*_) and transport rate constant (*k*). Note that *Γ*_*Pgp*_ was estimated based on the molar concentration of Pgp divided by the total surface area of membrane (i.e. lipids and Pgp molecules) per L of liposomal solution. Using the measured Pgp concentration of 0.138 mg/mL and MW of 170 kDa for hamster Pgp [3], molar concentration of Pgp was 0.81 μM. We estimated the surface area taken by lipids and Pgp in these liposomal preparations based on their concentrations of 0.138 mg/mL for Pgp and 1.38 mg/mL for lipids. To simplify the estimation of the total surface area occupied by lipid molecules, we based our calculation on the major lipid component, POPC (760 Da and surface area of 70 Å^2^) [59]. The total lipid surface area was halved in order to account for the fact that phospholipid molecules assemble into lipid bilayers resulting in an approximate surface area of ~ 383 m^2^/L for lipid bilayer. Likewise, we estimated the total surface area taken by Pgp molecules (170 kDa and surface area of 4900 Å^2^) [6] to be ~ 24 m^2^/L. Based on these values, the surface density of Pgp molecules was approximated by 0.81 μM/ (383+24) m^2^/L = 2 nmol/m^2^. Assuming a 50:50 inward: outward Pgp orientation in these liposomes and that only outward Pgp molecules could be activated in this system (since ATP cannot cross the membrane), the estimated surface density of outward Pgp molecules was about 1 nmol/m^2^.

**Fig 6:**
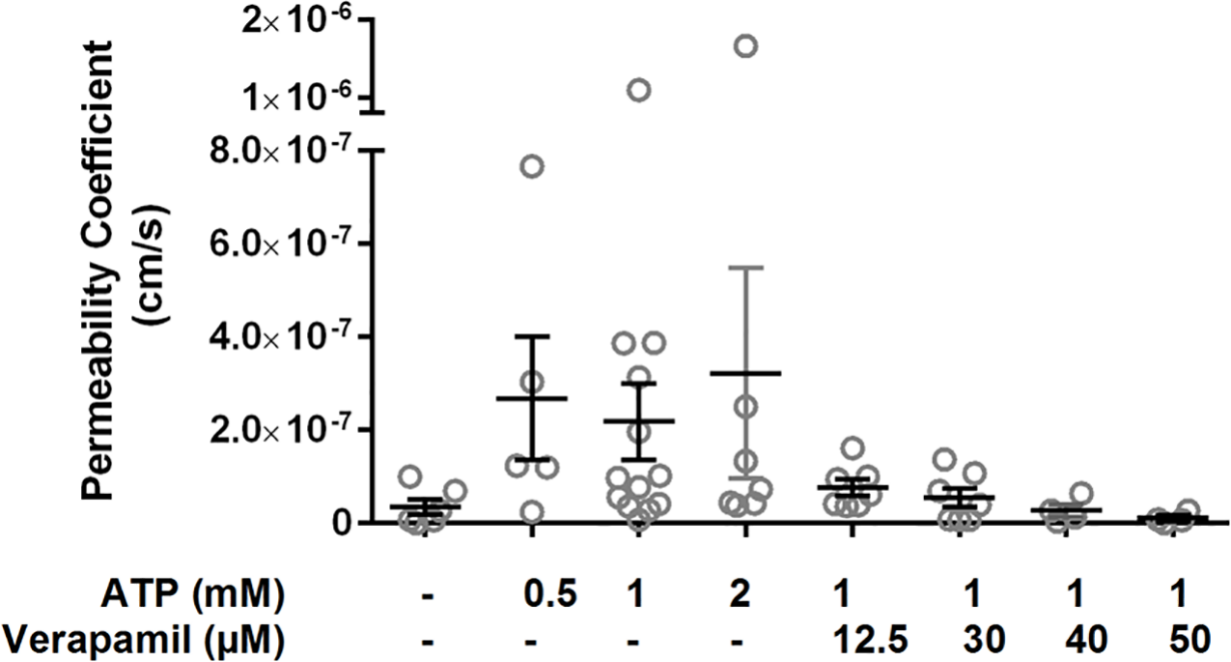
Permeability coefficient (*P*_*s*_) of Rho123 in Pgp-bearing giant liposomes under different conditions. According to one-way Anova, there was no significant difference in the value of *P*_*s*_ across all the examined conditions. Gray circles represent the data for individual liposomes from multiple independent experiments, and lines represent the mean and SEM (n = 4–13).

Fig 6 summarizes the obtained membrane permeability coefficients based on this analysis for all the transport activity assays performed. The permeability coefficients did not significantly differ among the examined conditions according to a one-way Anova test, and the average permeability coefficient for all conditions tested was 1.25×10^−7^ cm/s. Horger *et al* [38] have previously reported a *P*_*s*_ value of 10×10^−7^ cm/s for Rho123 across liposomal membranes bearing human Pgp and 1×10^−7^ cm/s for control liposomes (without Pgp), indicating an approximately 10-fold increase in membrane permeability due to the presence of Pgp in the membrane. In contrast, the results here showed the addition of Pgp on liposomal membranes had no significant impact on the membrane permeability of Rho123. Indeed, the *P*_*s*_ values measured in this study for liposomes with hamster Pgp were similar for all the tested conditions and were approximately 10-fold lower than permeability coefficients reported by Horger *et al*., suggesting better membrane integrity in these liposomes. The improved membrane integrity in the present liposomes may be attributed to the optimized cholesterol content in membrane composition as well as the lower Pgp surface densities on liposomes. Note that in a few cases throughout our studies, the measured *P*_*s*_ was higher than the abovementioned value of 10×10^−6^ cm/s, presumably due to possible uncontrollable membrane leakiness in the corresponding giant liposomes, which were subsequently excluded from further experimentation. The permeability coefficients together revealed that the observed influx of Rho123 into the giant liposome lumen was mainly due to the active transport by Pgp rather than the passive diffusion. This finding supports our previous analysis of the transport rates (1/s).

**Fig 7:**
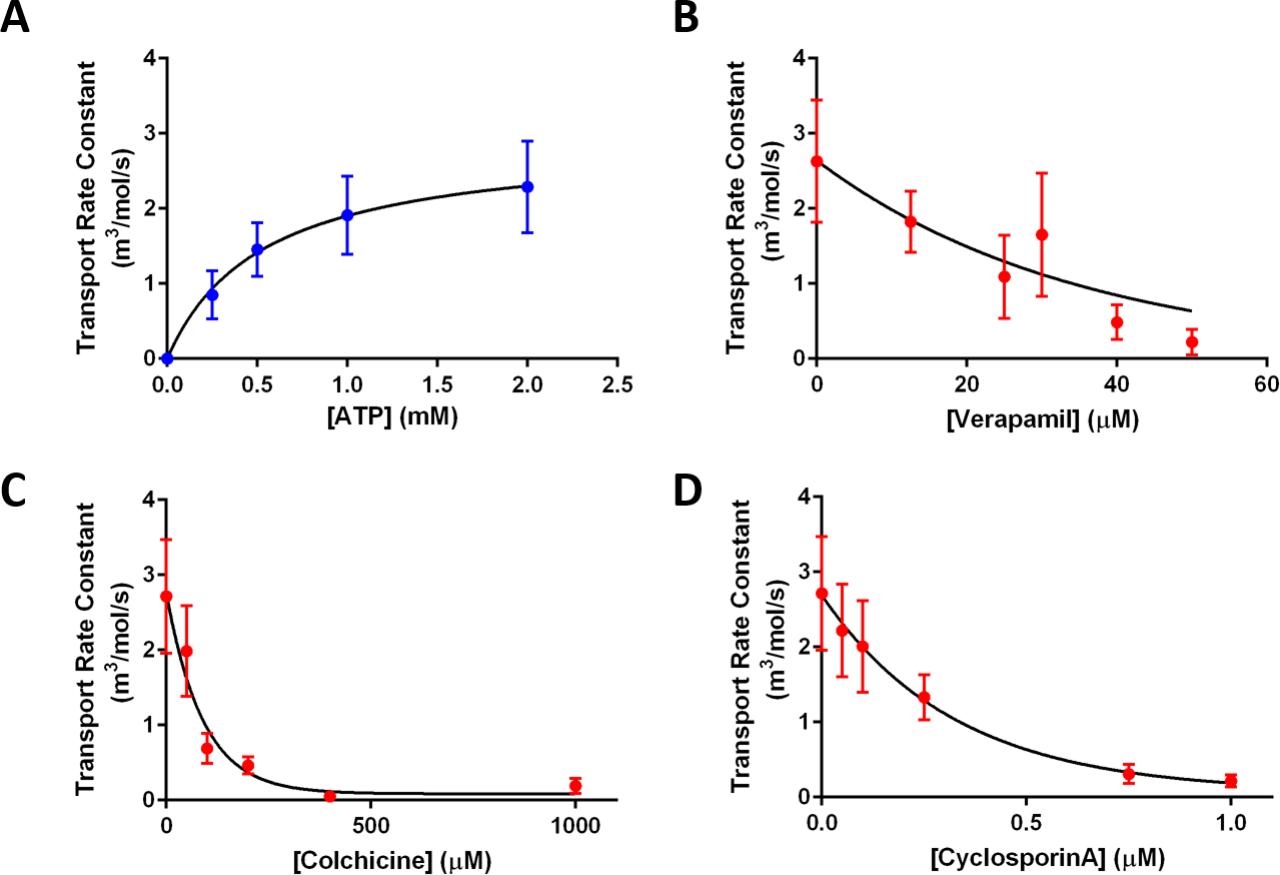
Evaluation of the transport rate constant (*k*) for Pgp-mediated transport of Rho123 under different conditions. (A) Transport rate constant as a function of ATP concentration. Mean and SEM are presented with n = 3–11 protroteoliposomes (from independent experiments) per condition tested. Data were fitted to Michaelis-Menten equation (*K*_*m*_ = 0.53 ± 0.66 mM). (B-D) Transport rate constants as a function of (B) verapamil concentration, (C) colchicine concentration, and (D) cyclosporin A concentration. Mean and SEM are presented with n = 4–12 from independent electroformations per condition tested. Data were fitted to a one-phase exponential decay model to estimate *IC*_*50*_ values of 25.2 ± 5.0 μM for verapamil, 61.8 ± 34.8 μM for colchicine, and 0.23 ± 0.09 μM for cyclosporin A.

From the non-linear fitting of time-dependent changes of *C*_*i*_*/C*_*o*_ to Eq 7, we also retrieved the transport rate constant, *k* (m^3^/mol/s) for the Pgp-mediated transport of Rho123. It should be noted that while the transport rates mentioned earlier encompassed both active and passive transport of Rho123 (i.e. active transport by Pgp and minor passive diffusion across membrane), the transport rate constant (*k*) obtained from data fitting to Eq 7 exclusively represents transport of Rho123 by active Pgp. However, these two parameters, transport rate and transport rate constant (*k*), exhibited similar trends in both ATP-dependent and verapamil-dependent transport activity. Indeed, as shown in Fig 7A, the transport rate constant increased with higher concentrations of ATP during transport activity following the Michaelis-Menten kinetic model. From the best fit of transport rate constants over the examined range of ATP to the Michaelis-Menten equation (Eq 2), *K*_*m*_ was determined to be 0.53 ± 0.66 mM. This value was not significantly different from the *K*_*m*_ of ATP retrieved from the transport rate (0.42 ± 0.75 mM). The slight increase in *K*_*m*_ based on the latter analysis may be due to the fact that this analysis separated the Rho123 passive diffusion from its Pgp-mediated transport. This separation could shift, the maximal transport rate constants to right, corresponding to higher ATP concentrations than that from the first analysis, in which the passive diffusion and Pgp-mediated transport of Rho123 were not separated.

Likewise, the transport rate constants with Pgp inhibitors in Fig 7B–7D showed comparable trends to that of transport rates in Fig 5B–5D. We also fitted the data in Fig 7B–7D to a one-phase exponential decay model as we did for data in Fig 5B–5D. Based on these curve fittings, *IC*_*50*_ values of the three examined inhibitors were determined to be 25.2 μM, 61.8 μM, and 0.23 μM for verapamil, colchicine, and cyclosporin A, respectively. These *IC*_*50*_ values did not significantly differ from those estimated earlier based on transport rates (Fig 5B–5D). There was, however, a leftward shift in *IC*_*50*_ values for verapamil and colchicine. This shift may again be attributed to the separation of passive diffusion from the active transport, which was not considered in our earlier analysis. In that analysis, the contribution of passive diffusion to the overall Rho123 transport was considered negligible. That contribution could, however, lead to an underestimation of the effect of inhibitors (i.e. higher *IC*_*50*_ values).

This work presents the evaluation of transport activity of hamster Pgp in single giant proteoliposomes, for the first time. We initially optimized the dehydration/rehydration/electroformation steps to minimize the protein activity loss. In order to eliminate the effect of variation in preparation steps on Pgp activity, we then applied our optimized procedure consistently throughout this study. However, day-to-day variation in experimental conditions such as temperature and humidity fluctuations are reflected in the presented data as, for instance, each data point in ATPase activity assay represents the normalized ATPase activity of batches of giant liposomes from multiple independent preparations (i.e. dehydration and rehydration events). Similarly, each data point for transport activity is from multiple independently prepared batches of liposomes from different days.

In conclusion, the applied electroformation technique allowed studying transport activity of Pgp efflux pump in individual liposomes using confocal microscopy. The ATPase activity of Pgp in giant liposomes was comparable to that in small liposomes, suggesting that the applied electroformation method had a minimal impact on this protein. We applied this platform to study the effect of ATP concentration and the presence of different Pgp inhibitors on Pgp-mediated transport of Rho123. Real-time monitoring of Rho123 fluorescence in liposomal lumen provided visual confirmation for Pgp transport activity, which is not possible in commonly used small proteoliposomes. Facile manipulation of lipid composition on giant proteoliposomes allowed for efficient optimization of membrane composition without the need for multiple reconstitution steps. This feature will be particularly beneficial for studying Pgp in different lipid environments using minimal amounts of purified protein. Two approaches were applied to determine the kinetic parameters for Pgp transport activity under the examined conditions and the estimated kinetic parameters showed the same trend as previous studies. This approach may be further applied for examining different aspects of Pgp functionality under well-defined conditions and may hence, facilitate the development of effective strategies to overcome MDR.

## Supporting information

S1 Fig Controlling the final lipid composition of giant liposomes without repeating Pgp reconstitution. (A) Phase contrast and fluorescence images of a representative giant liposome, electroformed from an equal volume mixture of NBD-PE and Pgp containing small proteoliposomes and Rho-PE (rhodamine) containing small liposomes. Phase contrast image shows the membrane unilamellarity in this giant liposome, and green and red fluorescence images demonstrate the even distribution of NBD-PE and Rho-PE on this liposome. (B) Fluorescence intensity of giant liposomal membranes from various volume mixtures of the Pgp-bearing NBD-labeled small proteoliposomes and Rho- labeled small liposomes. Data points represent mean intensities and error bars represent SEM (n = 10–12) Changes in green and red fluorescence signals in various mixtures of small liposomes suggest that the final lipid composition on the giant liposomes may be regulated via mixing of small liposome populations.
(TIF)

S2 Fig Evaluation of lamellarity of the giant liposomes. (A) Confocal images of giant unilamellar vesicles without Pgp (Control GUV), and giant proteoliposomes harboring Pgp (Pgp GUV) that were applied for Rho123 transport studies, and multilamellar liposomes show clear differences in Rho-PE distribution and intensity between unilamellar and multilamellar liposomes. (B) Rho-PE fluorescence intensities normalized by the size of giant liposomes. Control GUV and Pgp GUV showed similar fluorescence intensities while multilmellar liposomes displayed significantly higher intensities. Bars represent mean normalized intensities and error bars represent SEM (n = 28, 58, and 15 respectively). One-way Anova test was performed to validate the statistical significance.
(TIF)
