# Supplementary figures and images for: Reconstitution and functional studies of hamster P-glycoprotein in giant liposomes
Source: PLoS One. 2018 Jun 18;13(6):e0199279. doi: 10.1371/journal.pone.0199279 (PMC6005519; doi:10.1371/journal.pone.0199279)

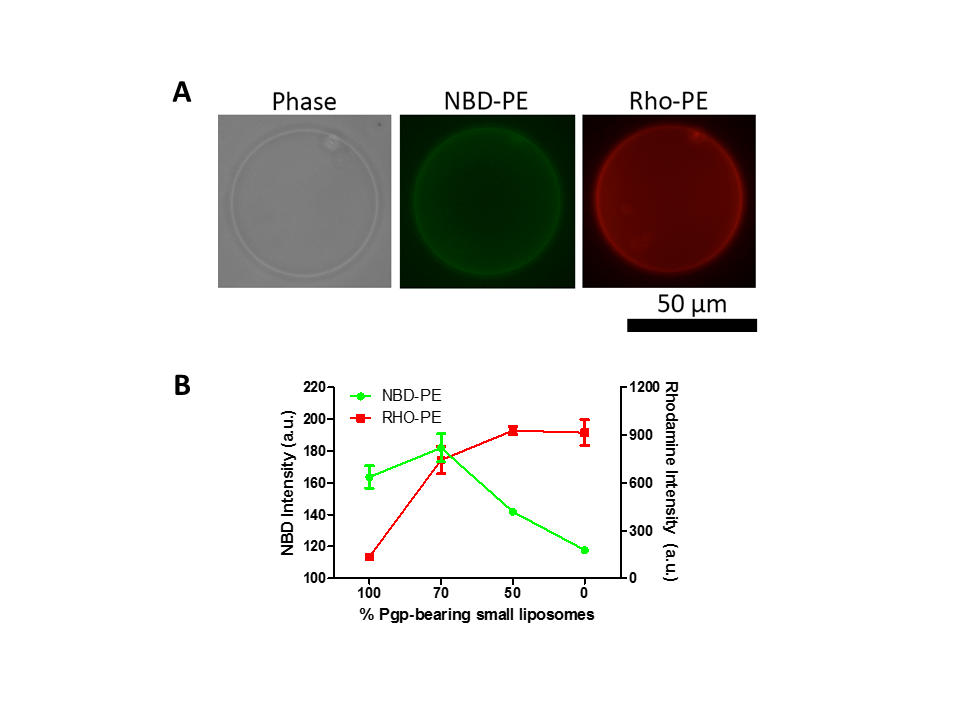

Supplement: S1 Fig — (A) Phase contrast and fluorescence images of a representative giant liposome, electroformed from an equal volume mixture of NBD-PE and Pgp containing small proteoliposomes and Rho-PE (rhodamine) containing small liposomes. Phase contrast image shows the membrane unilamellarity in this giant liposome, and green and red fluorescence images demonstrate the even distribution of NBD-PE and Rho-PE on this liposome. (B) Fluorescence intensity of giant liposomal membranes from various volume mixtures of the Pgp-bearing NBD-labeled small proteoliposomes and Rho- labeled small liposomes. Data points represent mean intensities and error bars represent SEM (n = 10–12) Changes in green and red fluorescence signals in various mixtures of small liposomes suggest that the final lipid composition on the giant liposomes may be regulated via mixing of small liposome populations. (TIF) [file pone.0199279.s001.TIF]

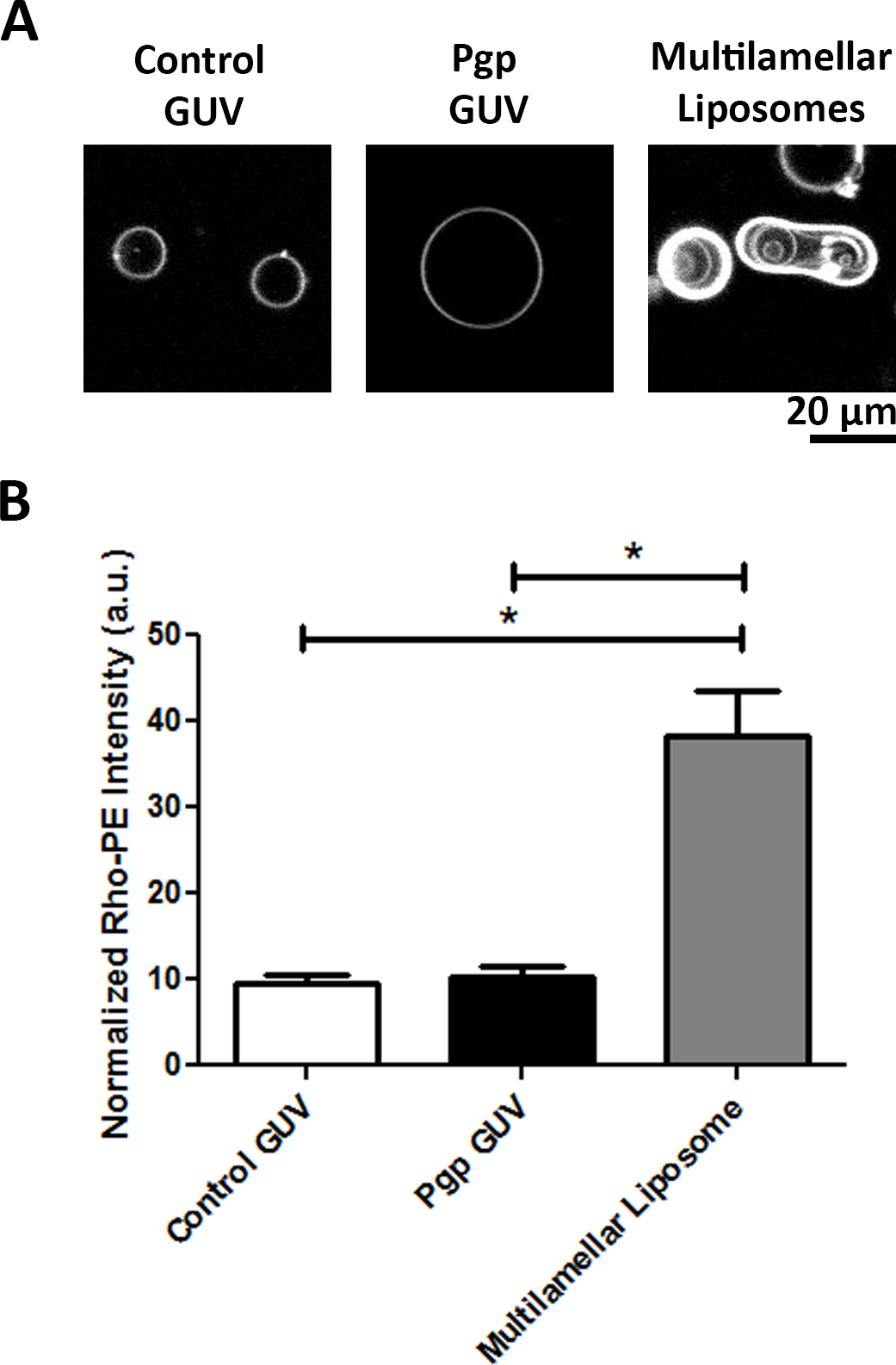

Supplement: S2 Fig — (A) Confocal images of giant unilamellar vesicles without Pgp (Control GUV), and giant proteoliposomes harboring Pgp (Pgp GUV) that were applied for Rho123 transport studies, and multilamellar liposomes show clear differences in Rho-PE distribution and intensity between unilamellar and multilamellar liposomes. (B) Rho-PE fluorescence intensities normalized by the size of giant liposomes. Control GUV and Pgp GUV showed similar fluorescence intensities while multilmellar liposomes displayed significantly higher intensities. Bars represent mean normalized intensities and error bars represent SEM (n = 28, 58, and 15 respectively). One-way Anova test was performed to validate the statistical significance. (TIF) [file pone.0199279.s002.tif]
